# Dermatological complications due to post‑COVID‑19 syndrome: A systematic review

**DOI:** 10.3892/mi.2024.208

**Published:** 2024-11-26

**Authors:** Beatriz Arely Cayón Figueroa, Wendy Mendoza Rojas, Daniel Tiburcio Jiménez

**Affiliations:** 1 Division of Internal Medicine, Hospital Regional ISSSTE León, León 37520, Guanajuato, Mexico; 2 Departament of Dermatology, Hospital of Specialties CMN XXI Century Dr Bernardo Sepúlveda, Mexico City 06720, Mexico; 3 School of Medicine, University of Colima, Colima 28040, Mexico

**Keywords:** post-COVID-19 syndrome, skin disorders, autoimmunity, hair loss

## Abstract

Almost 20% of patients affected by COVID-19 develop dermatological symptoms after recovery. This condition is termed as post-COVID-19 syndrome and is characterized by a state of hyperinflammation, as well as deregulations in the humoral response of CD8^+^ T-cells. Since there is no specific treatment for these injuries, the treatment of choice depends on the symptoms; thus, it is essential to provide a description of the type and nature of the injuries presented. In the present study, a systemic review was performed using the PICO strategy in the repositories of PubMed, Google Scholar, Embase and Scopus using the key words ‘POST-COVID and skin symptoms’. A total of 44 studies were included in the present systematic review. In these studies, the majority of subjects exhibited non-specific symptoms, commonly referred to as ‘skin rash’, affecting up to 27.4% of the population. According to the studies, a maximum prevalence of 50% of alopecia was observed among the affected subjects. Additionally, several studies reported the prevalence of other lesions, including pruritus (25%), subcutaneous nodules (12%), dermatitis (9.4%), edema (9%) and pigmentation changes (6%). Dermatological lesions associated with autoimmunity were also identified, with these findings being more pronounced among females and in patients with a history of severe COVID-19. Finally, several studies investigated the presence of autoantibodies, revealing a maximum prevalence of 41% for autoantibodies targeting the epidermis. Specifically, the prevalence was 12.9% for the Wuhan variant, 5.7% for the Alpha variant and 5% for the Delta variant. Although mostly benign, post-COVID-19 syndrome produces marked dermatological alterations for hair health, mainly among females. This, together with the development of lesions with an autoimmune component, constitutes an emerging therapeutic need.

## Introduction

The COVID-19 pandemic had severe repercussions on global health; currently, this infection is known for its ability to harm any organ or system in the body (1). As with other viruses (2), the symptoms it causes can become chronic (3). Indeed, due to the success of vaccination efforts, it is estimated that 70% of COVID-19 infections will have a greater long-term impact than during the acute phase of the infection (4). The persistence of symptoms following a COVID-19 infection is known as post-COVID-19 syndrome (5). This condition is characterized by the presence of symptoms for at least 2 months following the initial infection (6,7). The emergence of this syndrome does not depend on the duration of the primary infection (8), but rather on factors, such as age and the number of symptoms during the acute infection (9). The overall prevalence of post-COVID-19 syndrome has been calculated at 41.79%, although significant variations exist across different populations (10). Due to the ability of the virus to cross mucosal barriers (11), post-COVID-19 syndrome can manifest in the cells and structures of the skin (12). Potential lesions associated with COVID-19 have been classified into six groups, listed in order of prevalence: Acral (51%), vesicular (15%), urticarial (9%), morbilliform (9%), petechial (3%), livedo reticularis (1%) and other types (15%) (13). The mechanisms related to these lesions include the excessive production of inflammatory cytokines and stress hormones with subsequent damage to the production of proteoglycans in the extracellular matrix (14), as well as dysregulations in the humoral response of CD8+ T-lymphocytes (15) and residual effects of post-traumatic stress (16). However, the dermatological lesions observed in post-COVID-19 syndrome may differ from those observed during the acute phase of infection. Given that there is no specific treatment for dermatological lesions caused by post-COVID-19 syndrome, the treatment of choice depends on the symptomatology, thus rendering it essential to describe the type and nature of the lesions presented (17). For this reason, in the present study, a systematic review of the dermatological lesions and symptoms presented during post-COVID-19 syndrome was conducted to detail the clinical spectrum of this condition and guide the therapeutic needs of those affected.

## Data and methods

**Figure 1:**
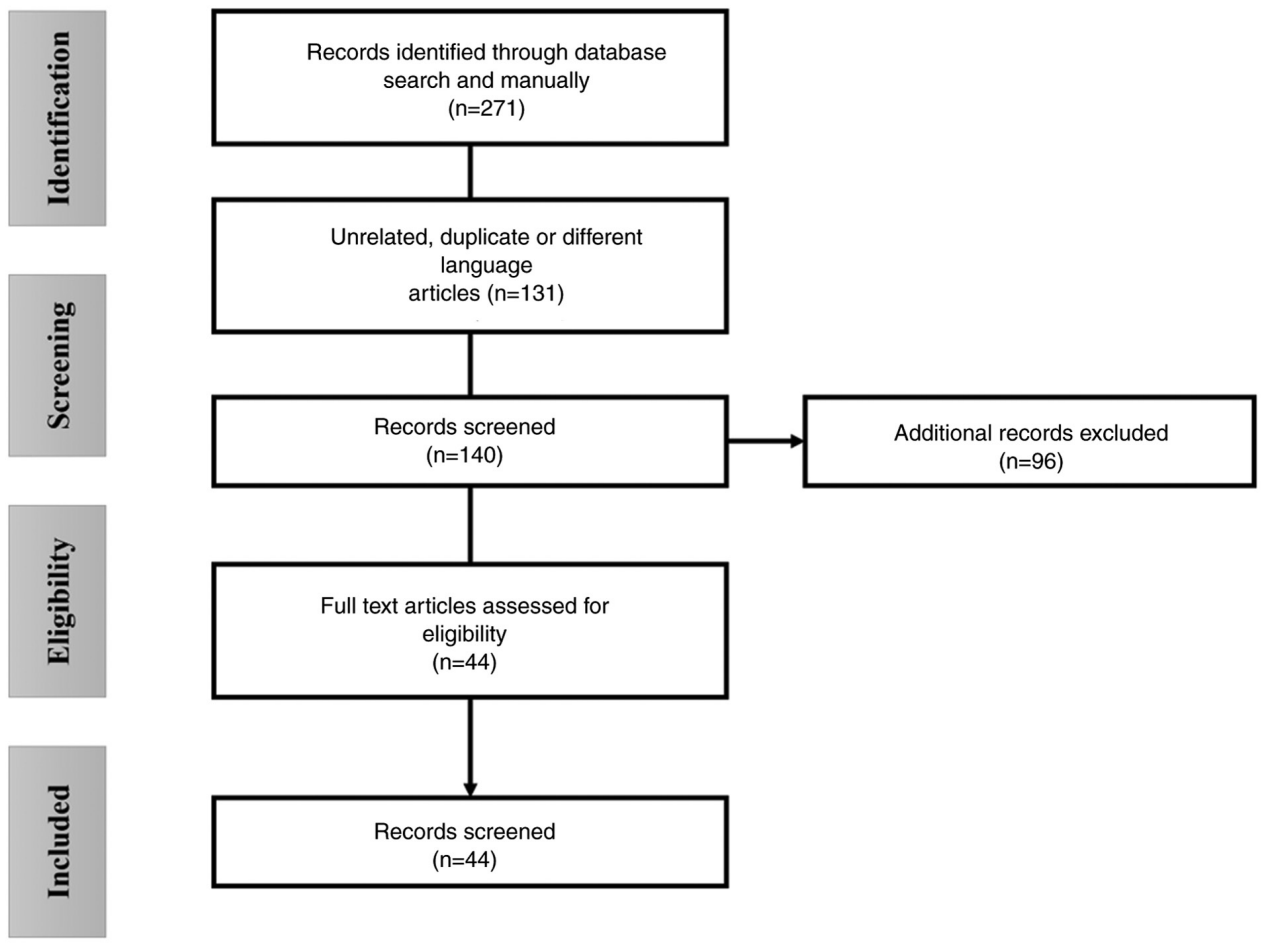
Flowchart of the study strategy for the selection of related articles.

Using the PICO strategy, a systematic search was conducted in the repositories of PubMed, Embase, Scopus and Google Scholar, utilizing the following key words: ‘Long-Covid Syndrome and skin manifestations’ OR ‘POST-COVID syndrome and cutaneous manifestations’ OR ‘Post COVID and Skin’. Only original multicentric articles, such as prospective cohort studies, retrospective cohort studies, case series and case reports published between January, 2020 and January, 2024, written in English or Spanish, involving patients diagnosed with post-COVID-19 syndrome and documenting clinical skin manifestations or cutaneous appendages, were included. Studies, such as systematic reviews, meta-analyses, expert consensus, pre-experimental studies and *in vitro* studies were excluded, as well as studies with unavailable content or imprecise information about symptoms and follow-up. Initially, a total of 271 candidate articles were found, which were reviewed in parallel by two reviewers through the reading of their abstracts and titles. Following the consensus of the reviewers, the results of this analysis were organized in a database, summarizing the characteristics of each record. A total of 119 studies were discarded due to unrelated information to the interests of the present systematic review, another 96 for belonging to other types of studies, one article was excluded for being in a language other than Spanish or English, and 12 studies were excluded for duplications, resulting in 44 eligible articles (Fig. 1).

In the 44 selected articles, data corresponding to the research question were collected, along with information regarding the number of participants, presented dermatological symptoms and follow-up period, among other relevant findings. Of the included articles, a total of 21 studies were prospective cohort studies, while four studies were retrospective cohort studies. Of note, one study was ambispective, and another was a case-control type study. Additionally, nine studies were cross-sectional, one study was a case series, and seven studies were case reports. A list of the included studies is presented in Table I.

## Results

**Figure 2:**
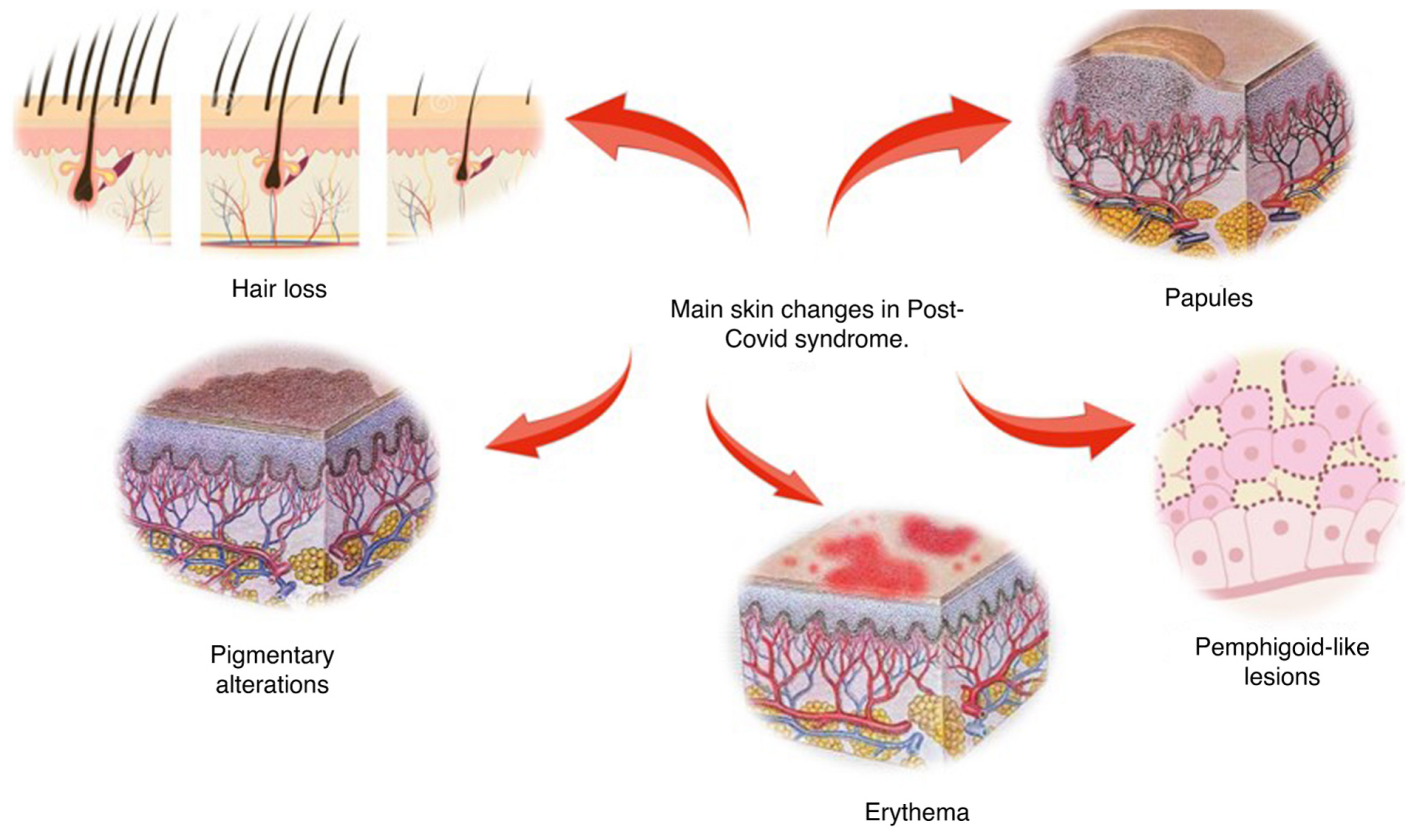
Main skin changes in post-COVID-19 syndrome.

Taken together, the analyzed studies compiled findings from 626,128 patients screened for dermatological symptoms secondary to post-COVID-19 syndrome. In the majority of the records, the findings occurred in a population that was previously healthy. In five studies, the target population was notable for the presence of chronical pathologies, psychiatric comorbidities, or a history of autoimmunity. Furthermore, one study focused on describing dermatological symptoms due to post-COVID-19 syndrome in the pediatric population. The average follow-up period and the onset of symptoms within the analyzed were ~7 months, with a minimum follow-up of 6 weeks following the diagnosis of COVID-19 and up to a maximum of 12 months post-diagnosis. The most prevalent dermatological symptoms in the population are detailed in specific sections below. Additionally, Fig. 2 provides a graphical representation of examples of these conditions.

### Non-specific dermatological manifestations

Among the studies analyzed, 14 articles described non-specific cutaneous symptoms as the main finding. These symptoms were described as ‘skin rash’, ‘dermatological alterations’ and ‘cutaneous symptoms’, without specifying the nature or characteristics of these alterations (18-31). The prevalence of this condition varied among the articles, with the majority of studies indicating a percentage ranging from 5 to 11% of the population. Of note, one study reported a minimum of 1.2% (30), and another reported a maximum prevalence of 27.4% (29) in the population. Additionally, three studies highlighted the female sex and hospitalization as risk factors for presenting these symptoms with greater severity (22,29,31).

### Symptoms associated with hair health

In addition to the non-specific cutaneous symptoms, a total of 13 studies described a certain degree of affliction in hair health. These conditions ranged from a decrease in hair density and thinning of the follicle to pronounced persistent alopecia lasting for months, with these issues enduring for an average period of 3 months following acute COVID-19 infection. The prevalence of this hair loss demonstrated a high variability, manifesting in as few as 2% of the population (32) to >50% of study subjects (33), with the majority of the studies indicating a prevalence between 7 to 35% (34-43). In two studies, the severity of alopecia was linked to both the female sex (32) and to the severity of the initial COVID-19 infection (43).

### Skin lesions

On the other hand, six studies described well-defined skin lesions with distinct characteristics. In two studies, the presence of papular lesions with irregular borders and erythematous features was described (44,45). In two other studies, the presence of subcutaneous nodules was documented (46,47), one of which also reported the occurrence of pruritus in 25% of the population, as well as the formation of blisters and subcutaneous nodules in 12% of subjects, edema in 9%, pigmentary changes and vesicle formation in 6% (47). Additionally, one study mentioned ecchymosis associated with vasculitis (48), and another described pemphigus vulgaris-like lesions (49).

### Dermatitis/associated pathologies

In six studies, cases of dermatitis and pathologies that appeared concomitantly in the subjects analyzed were presented. In one study, ~9.4% of the population exhibited dermatitis with a significant inflammatory component (50). Additionally, two studies mentioned the worsening of pre-existing pathologies related to autoimmunity, such as the clinical reactivation of psoriasis (51) and dermatomyositis (52), as well as the reactivation of the Epstein-Barr virus with the development of characteristic herpetic lesions (53). Furthermore, two cases of mucormycosis in immunocompetent patients were reported (54,55).

### Additional dermatological findings

A total of four studies focused on uncommon alterations. In two studies, antibodies against the epidermis were found in up to 41% of the affected subjects (56,57). On the other hand, one study analyzed the prevalence of cutaneous symptoms based on the viral variant detected during the primary infection, with a prevalence of 12.9% for the Wuhan variant, 5.7% for the Alpha variant and 5% for the Delta type (58). Finally, one study reported the presence of ‘COVID toes’, a finding where the skin of one or more toes exhibits edema and bright erythema that gradually turns violet, potentially showing violaceous brown spots, in 1.7% of its population (59).

### Possible pathophysiological mechanisms involved in dermatological symptoms in post-COVID-19 infection

Some of the studies reviewed described potential pathophysiological mechanisms involved in the onset of dermatological symptoms during the post-COVID-19 state. First, the cytotoxic potential of the coronavirus was suggested as a possible contributing factor through molecular interactions with angiotensin-converting enzyme 2 (ACE2) receptors present on the host cell surface, which are abundantly expressed in the skin (60). The interaction between SARS-CoV-2 and this receptor induces endothelial dysfunction characterized by localized hyperinflammation, vasculitis, deposition of complement proteins, and the subsequent development of skin and appendage lesions (61).

Furthermore, the persistence of this inflammatory state would increase the production of specific interleukins (IL), such as IL-6 and IL-4. The former has been linked to the loss of immune regulation that normally maintains hair follicles in an immune-privileged state, thereby ensuring their normal growth (62). Elevated levels of IL-4, on the other hand, promote keratinocyte apoptosis (63). These alterations, combined with the overexpression of matrix metalloproteinases 1 and 3, may represent the underlying mechanism responsible for symptoms, such as hair loss. This is due to the fact that they can modify the cellular microenvironment at the base of the hair bulb, thereby promoting the initiation of the catagen phase in the hair growth cycle (43).

Additionally, the studies included in the present systematic review describe mechanisms related to alterations in the hypothalamic-pituitary axis, generation of anti-epidermal antibodies and mitochondrial dysfunction in epidermal cells. These mechanisms have been previously identified as contributors to long-term dermatological symptoms, not only in COVID-19, but also in other viral infections, suggesting they may represent a common pathogenic pathway (64,65).

### Clinical implications of the findings for dermatological practice

According to the analyzed studies, dermatological symptoms following COVID-19 infection constitute one of the five major symptom clusters observed in recovered patients. Notably, these symptoms tend to appear at a later stage (22). The average prevalence of dermatological symptoms in affected individuals is 51.07%, primarily concerning hair-related conditions, which have been proven to be among the most challenging to treat due to their poor response to treatment (with a resolution rate <50%). Additionally, they are associated with a more prominent negative effect on the quality of life of patients and emotional well-being (19,29,36,37).

Moreover, the onset of these symptoms appears to follow a variable pattern, with manifestation occurring as early as 3 months post-acute phase, up to a maximum of 18 months in studies with longer follow-up periods. Notably, there is a possibility that these dermatological lesions may become chronic, as suggested by a study theorizing that dermatological manifestations following COVID-19 may persist indefinitely if left untreated (32) or may reappear periodically with increased severity (47).

The preceding statements hold significant implications for dermatological clinical practice. Given that skin lesions secondary to the post-COVID-19 condition tend to manifest at a later stage, establishing a link between a prior COVID-19 infection and these conditions can be particularly challenging. Furthermore, studies indicate a poor therapeutic response to these lesions, particularly in cases of hair loss, which is often perceived as a relatively minor issue by numerous healthcare professionals. However, this complication has emerged as having the greatest impact on the overall well-being of individuals, rendering their management a priority in dermatological care.

## Discussion

The present systematic review detailed the findings from studies focusing on post-COVID-19 syndrome and the dermatological conditions it causes. The literature review indicated a relatively limited number of records of dermatological alterations resulting from this syndrome. This scarcity may be due to the fact that such dermatological manifestations are not as apparent until the initial impact of the health emergency is mitigated (66). On the one hand, while the majority of studies reported only mild and non-specific skin lesions, other studies revealed two main findings: Firstly, an increase in cutaneous symptoms associated with hyperinflammation and immune dysregulations, and secondly, a significant prevalence of hair loss and alopecia in the affected population.

As regards the first finding, the included studies reported multiple cases of subjects whose pathology was associated with hyperinflammation and the development of autoimmunity, including the reactivation of autoimmune and viral diseases in a silent state, as well as infections by opportunistic pathogens in previously healthy individuals. Post-COVID-19 syndrome is characterized by the development of residual inflammation due to direct viral toxicity, as well as alterations in the regulatory function of T-lymphocytes (67). Studies focusing on this aspect have determined that after recovering from the acute phase of COVID-19, the virus can cause T-lymphocytes to exhibit autologous epitopes on their antigen identification cell receptors, thus creating an autoimmune environment, particularly in the female body (68).

Moreover, the excessive secretion of IL-6, D-dimer and lymphopenia produced during the primary COVID-19 infection increases the likelihood of developing post-COVID-19 syndrome (69). This is consistent with the findings that have been reported in previous research (70) and in the present systematic review, where females and subjects with severe COVID-19 infection presented more intense dermatological symptoms compared to the remainder of the population. In line with this finding, previous research has confirmed that some patients develop autoantibodies following COVID-19 infection, regardless of whether they have a history of autoimmunity or not (71). A proposed mechanism indicates the viral capacity to generate molecular mimicry with normal body structures (71). Thus, it is possible that the generation of antibodies against skin layers contributes to the development of skin lesions as indicated by some studies analyzed in this review (56,57).

As regards hair loss and alopecia, these conditions were initially reported during the peak of the pandemic in Spain (72), and described as expected findings during the acute phase and convalescence of patients infected with COVID-19(73). At that time, these conditions were considered mild and reversible, as with other viral and bacterial infections, resulting from the alteration of hair growth cycles following exposure to persistent inflammation affecting the metabolism of keratinocytes and the dermal papilla (74).

However, in the case of post-COVID-19 syndrome, it has been reported that the onset of alopecia occurs more rapidly and lasts longer compared to other pathogens, suggesting direct damage to the hair follicle. This is due to the fact that ACE2 receptors, used by COVID-19 for viral entry, are present in both mesenchymal cells of the hair follicle and its basal layers (75). Additionally, it has been theorized that certain components of the hair follicle increase immunological reactivity against COVID-19, consequently leading to an increase in antigen-antibody interactions in this area, resulting in the degradation of the follicle root structures (76). Furthermore, it is considered that this localized toxicity generates intense oxidative stress, leading to the premature onset of the catagen phase of the hair follicle and hair apoptosis, as well as a reduction in anticoagulant proteins in the perifollicular vessels, which in turn facilitates the formation of microthrombi that reduce blood flow to the hair root, causing it to thin and become fragile (77).

Although hair loss is considered a benign symptom without real systemic repercussions, its impact on body aesthetics is crucial for the individual, particularly considering that, compared with other dermatopathies, the availability of treatments for this condition is limited (78). This renders hair loss one of the most critical dermatological effects of post-COVID-19 syndrome, given the high prevalence rate of this condition. Thus, the timely approach to this condition in subjects with risk factors, such as women and severe COVID-19 should be considered a priority in dermatological health matters.

Although the findings from the present systematic review are notable due to their clinical implications in dermatological practice, it is important to highlight the inherent limitations of the present study. Since the studies analyzed predominantly rely on reports provided by affected patients, there is a notable risk of human bias. The integration of technological tools, such as neural networks for the analysis of dermatological lesions in post-COVID-19 syndrome, may allow for more accurate data on the impact of this condition on dermatological health, as has been observed with other conditions (79,80). Therefore, the associations generated through these analytical tools could contribute to future studies identifying both the risk factors for symptom onset and the pathogenic pathways responsible for the lesions in post-COVID-19 syndrome.

In conclusion, the present systematic review found that the dermatological manifestations of post-COVID-19 syndrome are primarily hair loss and skin lesions associated with persistent inflammation and the development of autoimmunity, with the severity of the initial clinical presentation and female sex being risk factors for severity. Additionally, given that dermatological lesions in post-COVID-19 syndrome may present in a delayed manner, their diagnosis should always be considered in patients experiencing symptoms that cannot be explained by other dermatological conditions. Furthermore, due to the limited therapeutic success for some symptoms, such as hair loss, further studies are required to focus on comparing specific therapeutic interventions for these conditions.

## Figures and Tables

**Table I:** Summary of the included studies.

Authors	Type of study	No. of patients	Features	Findings	(Refs.)
Menges *et al*	P	442	7-Month follow-up	Non-specific dermatological symptoms in 11% of patients	(18)
Peghin *et al*	A	231	Healthy population	10% Non-specific symptoms	(19)
Peter *et al*	R	11,710	12-Month follow-up	5.6% of patients had non-specific rash reactions	(20)
Popa *et al*	R	80	Healthy population	Non-specific dermatological symptoms	(21)
Munblit *et al*	P	2,649	More severe effects in women	8% of the population with nonspecific symptoms	(22)
Subramanian *et al*	P	48,6149	Healthy population	Unspecified alterations in nails, hair and skin	(23)
Fischer *et al*	P	491	12 Months follow-up	Non-specific symptoms in 23%, worsening depending on the severity of the initial condition	(24)
Leth *et al*	P	49	Hospitalized patients	Non-specific skin manifestations	(25)
Ariza *et al*	CS	319	Psychiatrics comorbid	Unidentified skin symptoms	(26)
Moreno-Pérez *et al*	P	277	14 Weeks follow-up	8.36% of patients with non-specific skin manifestations	(27)
Dryden *et al*	P	2,410	Healthy population	Skin rash 3.9% per month and 1.9% after 3 months	(28)
Šipetić *et al*	CS	51	Women with more severe manifestations	Alterations in nails, skin and hair: 27.4% of patients in general	(29)
Raj *et al*	CS	691	9-Month follow-up	Non-specific skin symptoms in 1.2% of patients	(30)
Funk *et al*	P	8,642	Pediatric population	Skin rash 12.50%, 10.30% in non-hospitalized patients	(31)
Asakura *et al*	CCS	8,018	Japanese population	1.5% alopecia, rash, cutaneous chilblains	(32)
Alkeraye *et al*	CS	806	Mostly women	52.7% with alopecia, persistence after 3 months	(33)
Fernández-de-Las-Peñas *et al*	P	1,969	Healthy population	Hair loss (23.9%) and rash (12%), more affected in women	(34)
Jung *et al*	R	1,122	4-Month follow-up	8.9% skin rash, 9.4% with noticeable hair loss	(35)
Kayaaslan *et al*	P	1,092	Healthy population	1% non-specific symptoms, and hair loss	(36)
Hennig *et al*	P	15	45 days of follow-up	Moderate and severe alopecia in the entire population	(37)
Förster *et al*	P	1,459	Several months of follow-up	Skin lesions in 2.3%, alopecia in 9.2% of patients	(38)
Domènech-Montoliu *et al*	P	484	More severe in females and COVID-19 severe infection	Skin lesions in 5.1%, loss of hair density in 22.25% of subjects	(39)
Al-Aly *et al*	P	73,453	Healthy population	Hair loss and skin lesions in 7% of subjects	(40)
Peter *et al*	CS	12,503	12 Months of follow-up	Skin rash, hair loss in 7% of subjects	(41)
Saberian *et al*	CS	7,226	Iranian population	32% with hair loss, 8% with skin lesions	(42)
Rossi *et al*	CSR	14	3 Months of follow-up	Hair loss, worse results are associated depending on the intensity of COVID	(43)
Dumont *et al*	P	1,034	12 Weeks follow-up	Skin lesions and pruritus in the population	(44)
McMahon *et al*	P	330	20 to 70 days follow-up	Presence of erythematous papules	(45)
Weinstock *et al*	CS	136	Several months of follow-up	Development of skin lesions, nodules and alopecia	(46)
Bouwensch *et al*	P	160	Healthy population	Pruritus in 25%, blisters and nodules in 12%, Rash, edema in 9%, vesicles and pigmentary alterations in 6% of patients	(47)
Morris *et al*	CR	1	1-Month follow-up	Ecchymosis and vasculitis	(48)
De Medeiros *et al*	CR	1	Immunocompetent patient	Case of pemphigus vulgaris with autoimmune component	(49)
Bekaryssova *et al*	CS	193	Healthy population	9.4% of the population with dermatitis	(50)
Qureshi and Bansal	CR	1	Patient with previous autoimmunity disease	Reactivation of psoriasis after COVID-19, associated hyperthyroidism	(51)
Shimizu *et al*	CR	1	Healthy patient	Case of dermatomyositis and nonspecific rash	(52)
Gold *et al*	P	185	Healthy population	7 subjects with skin rash and cases of Epstein Barr virus reactivation	(53)
Gupta *et al*	CR	1	Healthy patient	Report of mucormycosis	(54)
Saad and Mobarak	CR	1	Immunocompetent patient	Cutaneous mucormycosis secondary to COVID-19	(55)
Ahsan and Rani	CR	1	Healthy patient	Skin rash, anti-COVID antibodies detected	(56)
Richter *et al*	P	59	12-Month follow-up	Anti-epidermis antibodies were found (41%)	(57)
Fernández-de-Las-Peñas *et al*	P	614	Different population cohorts	Skin rash according to variant: Wuhan 12.9%, Alpha 5.7%, Delta 5.0%	(58)
Kim *et al*	P	454	12-Month follow-up	‘COVID toes’ in 1.7% of the population	(59)

P, prospective cohort study; R, retrospective cohort study; CCS, case-control study; A, ambispective cohort study; CS, cross-sectional study; CSS, case-series report; CR, case report.

## Data Availability

The datasets used and/or analyzed during the current study are available from the corresponding author on reasonable request.
